# Effect of bolus materials on dose deposition in deep tissues during electron beam radiotherapy

**DOI:** 10.1093/jrr/rrae001

**Published:** 2024-02-08

**Authors:** Dong Kong, Jia Wu, Xudong Kong, Jianfeng Huang, Yutian Zhao, Bo Yang, Qing Zhao, Ke Gu

**Affiliations:** Department of Radiation Oncology, Affiliated Hospital of Jiangnan University, No. 1000, Hefeng Road, Wuxi 214122, China; Department of Radiation Oncology, Affiliated Hospital of Jiangnan University, No. 1000, Hefeng Road, Wuxi 214122, China; Department of Radiation Oncology, Affiliated Hospital of Jiangnan University, No. 1000, Hefeng Road, Wuxi 214122, China; Department of Radiation Oncology, Affiliated Hospital of Jiangnan University, No. 1000, Hefeng Road, Wuxi 214122, China; Department of Radiation Oncology, Affiliated Hospital of Jiangnan University, No. 1000, Hefeng Road, Wuxi 214122, China; Department of Radiation Oncology, Affiliated Hospital of Jiangnan University, No. 1000, Hefeng Road, Wuxi 214122, China; Pharmaceutical Department, Affiliated Hospital of Jiangnan University, No. 1000, Hefeng Road, Wuxi 214122, China; Department of Radiation Oncology, Affiliated Hospital of Jiangnan University, No. 1000, Hefeng Road, Wuxi 214122, China

**Keywords:** radiotherapy, electron beam, bolus materials, Monte Carlo, dose distribution

## Abstract

Several materials are utilized in the production of bolus, which is essential for superficial tumor radiotherapy. This research aimed to compare the variations in dose deposition in deep tissues during electron beam radiotherapy when employing different bolus materials. Specifically, the study developed general superficial tumor models (S-T models) and postoperative breast cancer models (P-B models). Each model comprised a bolus made of water, polylactic acid (PLA), polystyrene, silica-gel or glycerol. Geant4 was employed to simulate the transportation of electron beams within the studied models, enabling the acquisition of dose distributions along the central axis of the field. A comparison was conducted to assess the dose distributions in deep tissues. In regions where the percentage depth dose (PDD) decreases rapidly, the relative doses (RDs) in the S-T models with silica-gel bolus exhibited the highest values. Subsequently, RDs for PLA, glycerol and polystyrene boluses followed in descending order. Notably, the RDs for glycerol and polystyrene boluses were consistently below 1. Within the P-B models, RDs for all four bolus materials are consistently below 1. Among them, the smallest RDs are observed with the glycerol bolus, followed by silica-gel, PLA and polystyrene bolus in ascending order. As PDDs are ~1–3% or smaller, the differences in RDs diminish rapidly until are only around 10%. For the S-T and P-B models, polystyrene and glycerol are the most suitable bolus materials, respectively. The choice of appropriate bolus materials, tailored to the specific treatment scenario, holds significant importance in safeguarding deep tissues during radiotherapy.

## INTRODUCTION

The purpose of using bolus in superficial tumor radiotherapy is to counteract the dose build-up effect of radiation, thereby ensuring uniform and conformal dose distribution within the target area [1–3]. Prior research has predominantly emphasized the impact of air gaps between the bolus and the skin on superficial dose [4–7], often exploring techniques like 3D printing to minimize such gaps [2, 3, 8, 9]. In clinical practice, a wide array of materials, including water, petrolatum, superflab, super-Flex, silica-gel and polystyrene, are utilized as bolus materials [10]. The choice of bolus materials significantly influences the deposition of radiation dose in tissues due to variations in the interactions with different materials. Kyeong-Hyeon *et al*. conducted a study examining the relationship between the thickness of silica-gel bolus and the depth of maximum dose in a phantom exposed to electron beam irradiation and discovered that the thickness of silica-gel was ~1.06 and 1.07 times that of water for 6 MeV and 9 MeV electron beams, respectively [11]. In another study by Franca Ujah Okoh *et al*., it was observed that polyvinyl alcohol (PVA)-based bolus had a maximum impact of no more than 2.9% on the percentage depth dose (PDD) in a solid water phantom. The superficial dose remained consistent with the standard bolus, and the PVA content did not affect it [12]. Additionally, Hsu *et al*. investigated the impact of various bolus materials on skin dose reduction in conventional tangential fields and intensity-modulated radiation therapy (IMRT) fields through measurements [13]. They found that, when compared to Superflab, fine mesh, solid and large mesh aquaplasts reduced skin dose by 21, 11, 9%, and 22, 12 and 10%, respectively. These studies primarily focused on assessing the impact of bolus on radiation doses in superficial tissues. As cancer treatments have advanced, patients’ survival rates have improved, leading to an increased focus on the development of secondary primary cancers caused by radiation. Storm H H’s study provided evidence that secondary cancer resulting from cervical cancer radiotherapy often manifests late, with the risk continuing to increase over a period of more than 30 years [14]. Another study by Ruth *et al*. demonstrated that the risk of radiation-induced cancer does not return to normal levels over time [15]. Furthermore, Keehn *et al*.’s research confirmed that the risk of bladder cancer after prostate cancer radiotherapy, especially following brachytherapy, increases after 10 years, with a lower stage and higher grade observed [16]. For breast cancer radiotherapy patients, studies by Huang *et al*. [17] and Rubino *et al*. [18] have corroborated that radiotherapy can elevate the incidence of various tissue sarcomas, with high-dose radiation significantly increasing the risk of cancer. As a result, it holds tremendous significance to minimize the radiation dose to normal tissues surrounding the target area to address potential risks of secondary cancers in the long term.

The primary objective of this study is to compare the dose distributions of electron beams in deep tissues when different bolus materials, including water, polylactic acid (PLA), polystyrene, silica-gel and glycerol—commonly used in clinical practice, were employed. Electron beam phase space files (PSFs) from the Varian 2100CD medical linear accelerator, which underwent rigorous consistency testing and experimental verification prior to their inclusion [19], were acquired from the International Atomic Energy Agency (IAEA) website. Subsequently, the geant4 Monte Carlo software package was utilized to construct various treatment models and compute the dose distributions of electron beams in these models. The findings of this study are intended to serve as a valuable reference for clinicians and physicians when selecting appropriate bolus materials in clinical settings.

## MATERIALS AND METHODS

### Constructing treatment models

**Fig. 1:**
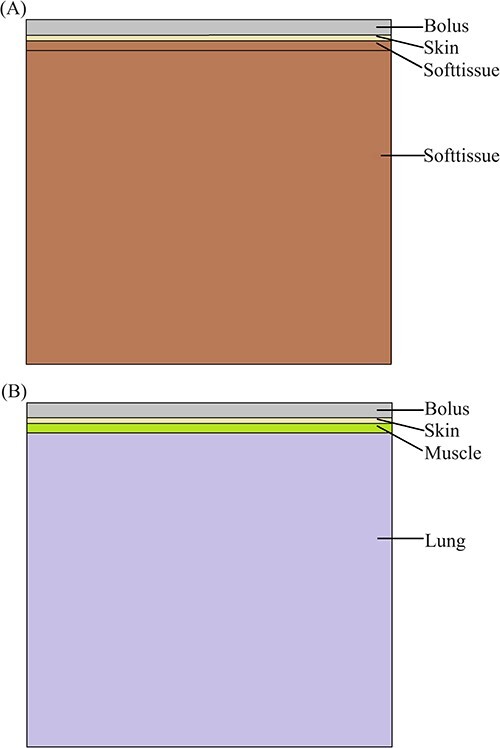
Treatment model. (**A**) Superficial tumor model; (**B**) Postoperative breast cancer model.

**Fig. 2:**
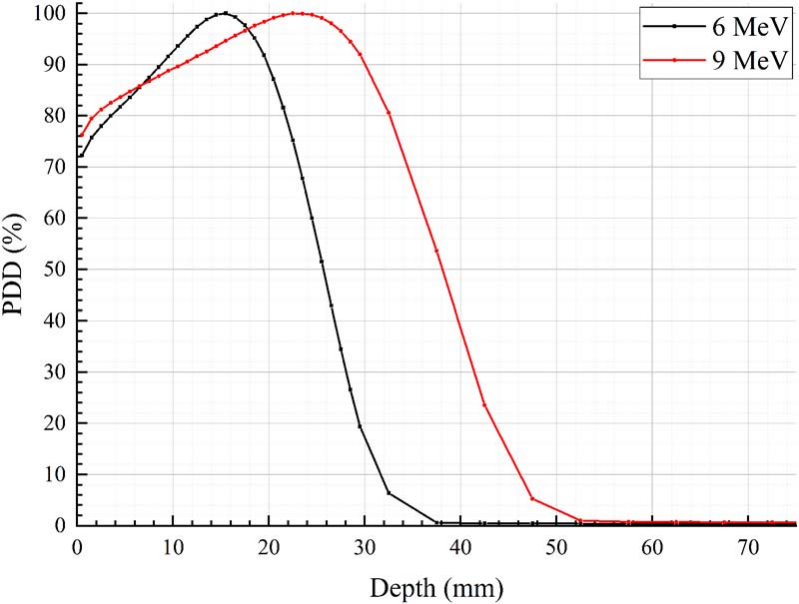
PDDs of 6 and 9 MeV electron beams in water.

In this study, two sets of treatment models were created: the S-T models designed for conventional superficial tumors, and the P-B models tailored for postoperative breast cancers. Each set of models is centered on the field central axis and has a size of 30  × 30 cm in the direction vertical to the beams. The top surface of the models is positioned at a distance of 100 cm from the accelerator virtual source (SAD = 100 cm). To illustrate the layers of the models, refer to Fig. 1. The first layer in each model represents the bolus. The material used for the bolus can be water, PLA, polystyrene, silica-gel or glycerol. The second layer represents the skin with a uniform thickness of 0.3 cm across all models. In the S-T models, both the third and fourth layers consist of soft tissue. However, in the P-B models, the third layer is composed of muscle, while the fourth layer is lung tissue. Considering that the second and third layers correspond to the tumor and potential areas of invasion, electron beam radiotherapy is necessitated, while the fourth layer represents the adjacent normal tissue following treatment. The thicknesses of the third layer are set at 0.5 , 1.0 or 1.5 cm, respectively. By considering the PDDs of 6 and 9 MeV electron beams in water (refer to Fig. 2) alongside the thickness of the third layer in the model, Table 1 offers the electron beam energy and corresponding bolus thickness relevant to the third layer’s depth. This specific selection is designed to ensure optimal dosage in the pre-irradiation area while minimizing the exposure of subsequent normal tissue to radiation. This approach aligns with the established principles and methodologies for selecting electron beam energy and bolus thickness in clinical practice. The fourth layer remains constant at 30 cm. To record the dose depositions of the electron beams, 1.0  × 1.0  × 0.1 cm voxels are placed along the field central axis within the models. For comprehensive information on the basic physical parameters of the materials utilized in the models, please refer to Table 2.

**Table 1:** Parameters of treatment models

Thickness of layer 3 (cm)	Electron beam energy (MeV)	Thickness of bolus (cm)
0.5	6	1.0
1.0	6	0.5
1.5	9	1.0

**Table 2:** Physical information of materials in the models

Materials	Elemental composition (mass fraction (%))	Density (g/cm^3^)	Electron density (N_A_/cm^3^)	Effective atomic number^a^
Water	H (11.2); O (88.8)	1.0000	0.5549	7.4923
Polystyrene	H (7.8); C (92.2)	1.0600	0.5699	5.7347
PLA	H (5.6); C (50.0); O (44.4)	1.1245	0.5929	6.8815
Glycerol	H (8.8); C (39.1); O (52.1)	1.2613	0.6847	6.9358
Silica-gel	H (8.1); C (32.4); O (21.6); Si (37.9)	1.1550	0.6226	10.6669
Skin	H (10); C (20.4); N (4.2); O (64.5); Na (0.2); P (0.1); S (0.2); Cl (0.3); K (0.1)	1.0900	0.5982	7.3926
Soft-tissue	H (10.5); C (25.6); N (2.7); O (60.2); Na (0.1); P (0.2); S (0.3); Cl (0.2); K (0.2)	1.0300	0.5678	7.3541
Muscle	H (10.2); C (14.3); N (3.4); O (71); Na (0.1); P (0.2); S (0.3); Cl (0.1); K (0.4)	1.0500	0.5773	7.5971
Lung	H (10.5); C (8.3); N (2.3); O (77.9); Na (0.2); P (0.1); S (0.2); Cl (0.3); K (0.2)	0.5000^b^	0.2756	7.6193

^a^The calculation formula can be found in 《Radiation Oncology Physics》 on page 34, for a mixture with the value of m taken as 3.4.

^b^The density of lung tissue ranges from 0.3450 to 0.7460 g/cm^3^, and in this study, it was taken as 0.5000 g/cm^3^.

### Electron beam sources and geant4 running conditions

In this study, the 6 and 9 MeV electron beams were adopted, due to their common clinical use. The initial particle source files for the electron beams with 10 × 10 cm fields from the Varian Clinac 2100CD medical linear accelerator were the PSFs obtained from the IAEA official website [20]. The operating system used was 64-bit Windows 10, and the C++ compiler employed was Microsoft Visual Studio Community 2019 (version 16.10.2). To simulate the transport process of the electron beams and calculate the dose deposition in the models, the geant4 Monte Carlo software package (version 10_07_p02) was utilized. The chosen physics model was LowE_Livermore with a cutoff range of 1 mm. For both 6 and 9 MeV electron beams, a total of 56 871 296 [21] and 56 197 810 [22] particles were sampled, respectively, from the initial PSFs. Subsequently, the particle transport process in air was simulated, and information on the particles reaching a plane with dimensions of 16 × 16  × 0.1 cm, positioned 3 cm above the bolus (i.e. at a distance of 97 cm from the virtual source) was recorded to generate corresponding new PSFs. These new PSFs were used as the particle sources for the subsequent calculations.

### Obtaining dose depositions in models

Geant4 was configured with the same settings as described in Section “Electron beam sources and geant4 running conditions”. This included adopting the LowE_Livermore physics model with a cutoff range of 1.0 mm. To reduce variance, geometric importance sampling was utilized, with different weightings assigned to various regions: weightings of 1, 10, 100 and 1000 were assigned to the peripheral space, the environment near the models, the models themselves and the voxels, respectively. The new PSFs generated above were used for each run, involving a total of 8 × 10^8^ events. For both the S-T models and P-B models, dose depositions and corresponding errors were separately calculated for different bolus materials. The calculated errors for the 6 and 9 MeV electron beams within the models were observed to be <0.5% within the depth range of 31–46 mm, and below 1% within the depth range of 95–160 mm.

### Data processing and drawing

The dose at the depth of maximum dose (*d*_max_) is commonly used as a reference when evaluating the impact of bolus on dose deposition [11, 12]. Hence, in the S-T models, the dose at *d*_max_ is chosen as the reference. Conversely, for the P-B models, where treatment extends to the anterior chest wall in cases of stage T3/4 breast cancer after modified radical mastectomy [23], the dose at the muscle interface (i.e. the voxel closest to the lung tissue) is taken as the reference. PDDs were obtained for models with bolus of different materials. The PDDs obtained with a water bolus were used as the standard, and the relative doses (RDs) with other bolus materials at the same position beyond the reference depth were calculated using Equation (1) (provided below). The RDs data were subjected to statistical analysis and plotted using the Origin 2018 64-bit software.


(1) \begin{equation*} RD=\frac{PDD_{M,d}}{PDD_{water,d}}\times 100\% \end{equation*}

In Equation (1), ${PDD}_{M,d}$ and ${PDD}_{water,d}$ represent the PDDs at depth d behind the reference depth when the bolus material is M and water, respectively.

## RESULTS

### RD differences in the S-T models

**Fig. 3:**
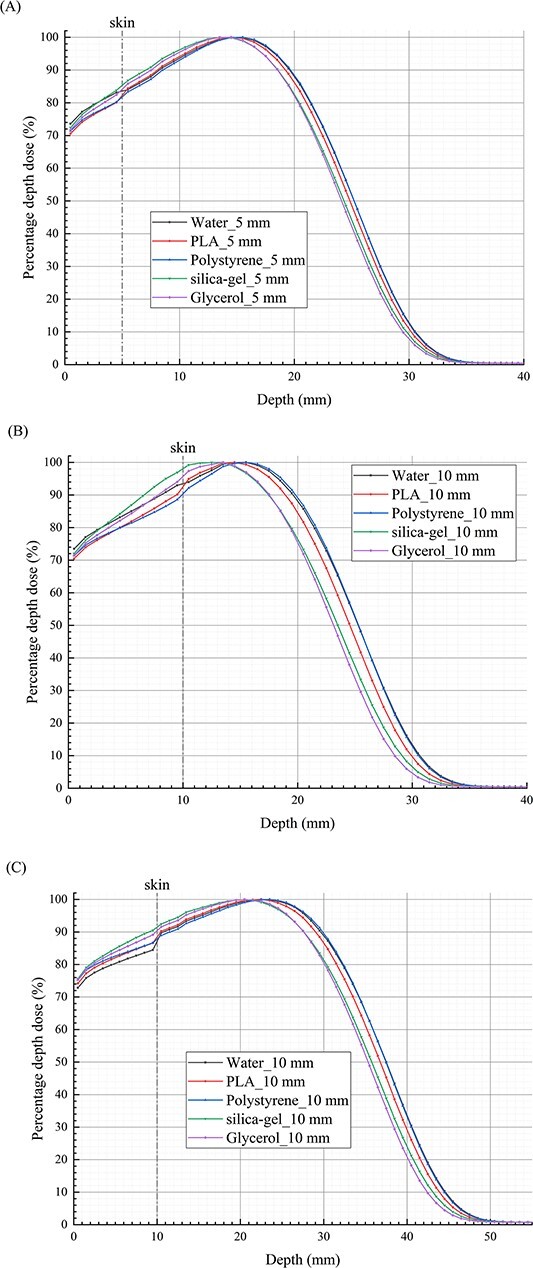
The distribution of PDDs in S-T models with boluses of different materials for electron beams. (**A**) PDDs for models with 0.5 cm thick bolus and 6 MeV electron beams. (**B**) PDDs for models with 1.0 cm thick bolus and 6 MeV electron beams. (**C**) PDDs for models with 1.0 cm thick bolus and 9 MeV electron beams. The *x*-axis is centered at bolus surface, and the reference line of skin is the boundary between skin and bolus.

**Fig. 4:**
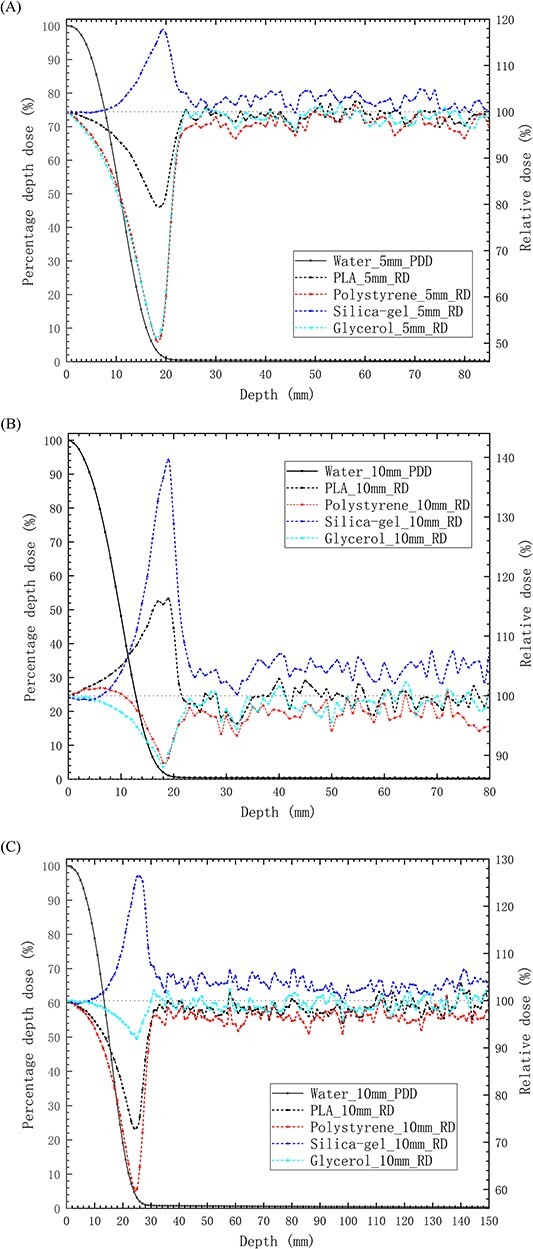
The distribution of PDDs and RDs beyond *d*_max_ in S-T models with boluses made of different materials for electron beams. (**A**) Results for models with 0.5 cm thick bolus and 6 MeV electron beams. (**B**) Results for models with 1.0 cm thick bolus and 6 MeV electron beams. (**C**) Results for models with 1.0 cm thick bolus and 9 MeV electron beams. The *x*-axis is centered at *d*_max_. The solid line represents the PDDs in the S-T models with a water bolus, while the dashed lines represent the RDs in the S-T models with boluses of other materials.

The *d*_max_ of the electron beams in tissue varies with different bolus materials. Figure 3 illustrates the PDDs distribution of electron beams in S-T models. To facilitate the comparison of RDs in the models with different bolus materials beyond *d*_max_, the *d*_max_ in the model with water bolus is set as the reference point, and the PDDs depth coordinates of other bolus materials need to be adjusted to ensure the consistency of *d*_max_ coordinates among different bolus materials. With reference to the PDDs in the S-T models with water bolus, it is observed that in regions where the dose decreases rapidly, the differences of RDs for models with bolus made of different materials gradually increase, then rapidly decrease until stabilizing. The impact of bolus materials on the dose distribution of electron beams beyond the *d*_max_ is dependent on both the bolus thickness and the electron beam energy, as depicted in Fig. 4. For 6 MeV electron beams, within the region ~1.8 cm after *d*_max_, the RD exhibits complex variations with changes in PLA bolus thickness. At a thickness of 5 mm, the RD is <100%, with a minimum value of 79.6%. However, at a thickness of 10 mm, the RD exceeds 100%, with a maximum value of 116.3%. On the other hand, for silica-gel bolus, the RD is consistently greater than 100% and increases proportionally with the increase in bolus thickness. The highest RD values are 117.3 and 139.6% for thicknesses of 0.5 and 1 cm, respectively. When using polystyrene and glycerol bolus materials, although the overall RDs are <100%, they increase with an increase in bolus thickness. Notably, the RD for a 1 cm polystyrene bolus even exceeds 100% within 1 cm after *d*_max_. Although the bolus materials influence the depth of the maximum/minimum RD values, the bolus thickness does not have a significant impact. For 1 cm thick boluses, as the electron beam energy increases to 9 MeV, the differences in RDs for models with bolus of different materials gradually increase within ~2.5 cm after *d*_max_, followed by a rapid decrease, ultimately stabilizing after around 0.6 cm. In the region where the doses decrease rapidly, the RD in the model with a PLA bolus is <100%, with a minimum value of only 72.9%. The trends of RDs for silica-gel, polystyrene and glycerol boluses remain consistent, but the magnitudes of change differ significantly. The maximum changes for these three materials are ~−13.3, −28.6 and 3.8%, respectively. The depths of the extreme points of the RDs are notably influenced by the electron beam energy when utilizing boluses of different materials. As shown in Table 3, it can be observed that the corresponding PDD values when the RDs reach the extreme points fall between 1.00 and 3.50%. For depths where the PDDs are below 1%, the differences in RDs among models with different bolus materials are minimal. In this scenario, the model with a silica-gel bolus exhibits the highest dose, followed by water bolus, PLA bolus and polystyrene bolus in descending order.

**Table 3:** The PDD value corresponding to the extreme values of RDs for different models with bolus of different materials (%)

Model	Energy (MeV)	Thickness (cm)	Bolus material			
			PLA	Polystyrene	Silica-gel	Glycerol
S-T	6	1.0	1.28	1.81	1.54	1.80
	6	0.5	1.51	1.78	2.22	1.80
	9	1.0	3.49	1.87	2.55	2.86
P-B	6	1.0	1.40	1.08	1.83	1.13
	6	0.5	2.16	1.01	1.74	1.37
	9	1.0	2.50	2.50	2.95	2.09

### RD differences in the P-B models

**Fig. 5:**
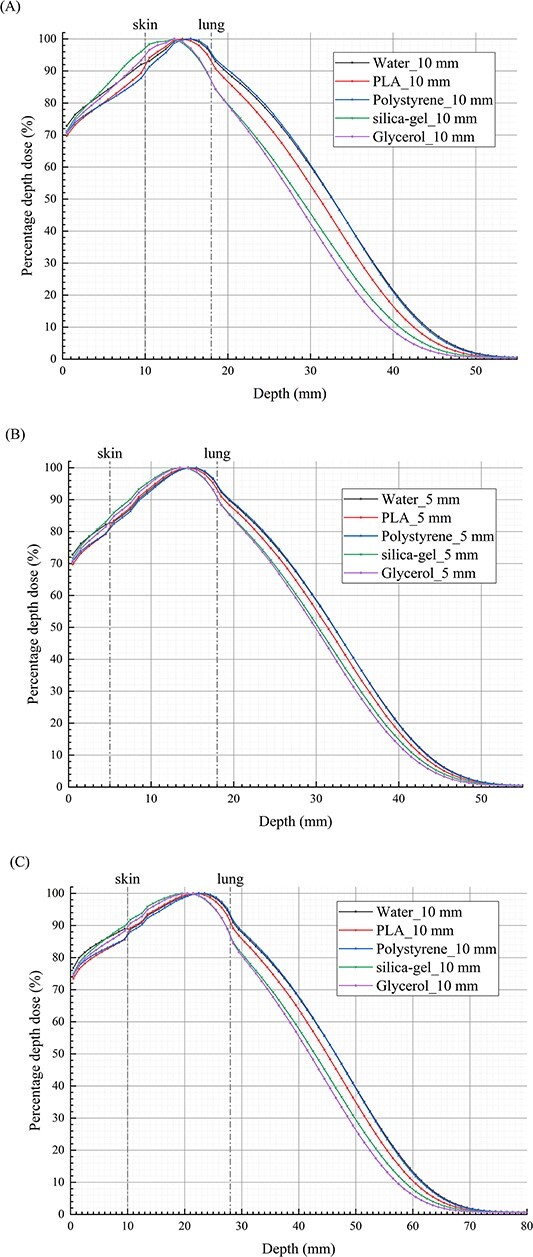
The distribution of PDDs in P-B models with boluses of different materials for electron beams. (**A**) PDDs for models with 0.5 cm thick muscle, 1.0 cm thick bolus and 6 MeV electron beams. (**B**) PDDs for models with 1.0 cm thick muscle, 0.5 cm thick bolus and 6 MeV electron beams. (**C**) PDDs for models with 1.5 cm thick muscle, 1.0 cm thick bolus and 9 MeV electron beams. The *x*-axis is centered at bolus surface, and the reference lines of skin and lung are the boundaries of skin-bolus and lung-muscle.

**Fig. 6:**
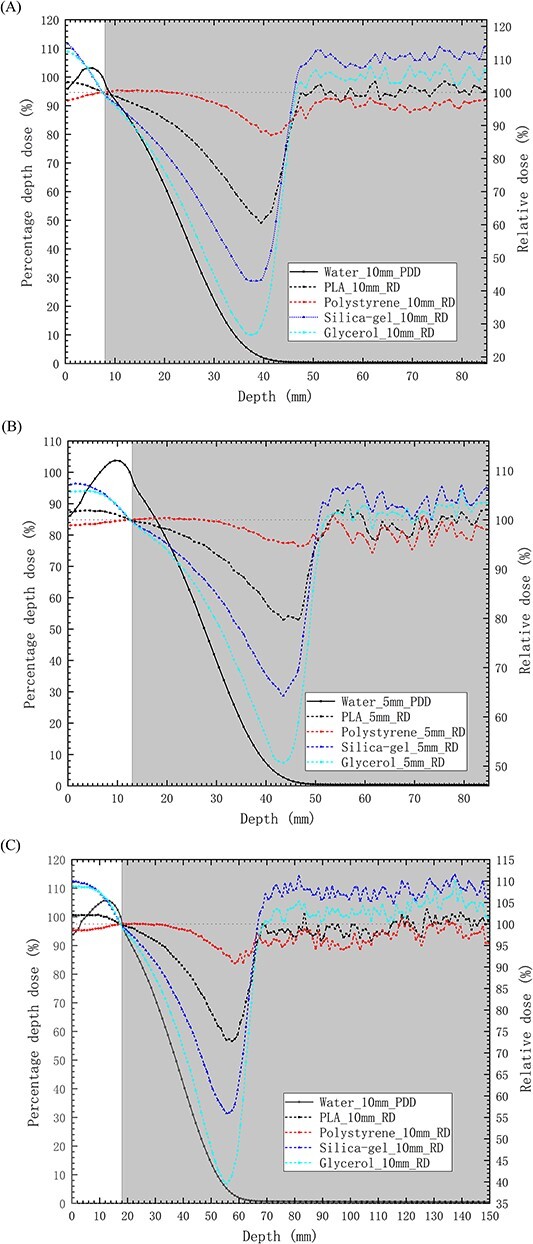
The distribution of PDDs and RDs beyond *d*_max_ in the P-B model with boluses made of different materials for electron beams. (**A**) Results for models with 0.5 cm thick muscle, 1.0 cm thick bolus and 6 MeV electron beams. (**B**) Results for models with 1.0 cm thick muscle, 0.5 cm thick bolus and 6 MeV electron beams. (**C**) Results for models with 1.5 cm thick muscle, 1.0 cm thick bolus and 9 MeV electron beams. The *x*-axis is centered at the skin surface. In the figures, the solid lines represent the PDDs in the P-B models with water boluses, while the dashed lines represent the RDs in the P-B models with boluses of other materials. The gray shaded areas represent the lung tissue.

Figure 5 depicts the PDDs distribution of 6 and 9 MeV electron beams in the P-B models. The figure highlights the distinct dose distributions observed in the skin, muscle and lung tissues with different bolus materials. To facilitate a comparative analysis of RDs in the lung tissue at equivalent doses to the muscle posterior boundary, each model employs the muscle posterior dose as the normalization point. In comparison to the water bolus, as the depth increases in the lung tissue, the RDs in models with boluses made of the other four materials initially decrease and then rapidly recover after reaching their minimum points, as depicted in Fig. 6. Regarding the PDDs in P-B models with a water bolus, for the same bolus thickness and electron beam energy, within regions where the dose decreases rapidly, the RDs in the model with a glycerol bolus exhibit the fastest decrease and reach the lowest value. Following this, the RDs for the silica-gel bolus, PLA bolus and polystyrene bolus consecutively increase in magnitude. Both the electron beam energy and bolus thickness impact the RDs in models with boluses, and in order of decreasing impact, the bolus materials are glycerol, silica-gel, PLA and polystyrene. For glycerol bolus, when the electron beam energy is 6 MeV and the bolus thickness increases from 0.5 to 1.0 cm, the minimum RD value decreases from 50.7 to 26.7%. When using 1.0 cm thick boluses, increasing the electron beam energy from 6 to 9 MeV results in the minimum RD value increasing to 39.5%. Conversely, for polystyrene bolus, the change in the minimum RD value is <10%. The depth at which the minimum RDs are achieved is influenced by the bolus materials and electron beam energy, rather than the bolus thickness. Notably, the faster the RDs drop, the shallower the depths of the minimum RD values. According to Table 3, in models with boluses of different materials, the RDs reach their minimum values when the PDDs in the lung drop to 1–3%. Subsequently, when the PDDs in these models drop to below 1%, the RDs tend to stabilize. In these regions, the highest dose in the lung tissue is achieved when using a silica-gel bolus, followed by using a glycerol bolus, water bolus, PLA bolus and polystyrene bolus.

## DISCUSSION

The use of bolus in electron beam therapy is known to effectively enhance the dose delivered to superficial tumors, thereby improving dose uniformity and conformity within the tumor region. However, the interaction between different materials and electron beams can significantly impact the dose distribution within tissues, leading to variations in the treatment outcome. In this study, we employed the Monte Carlo method to approximate the electron beam therapy scenario for general superficial tumors and postoperative breast cancer, utilizing the S-T models and P-B models, respectively. We compared the differences in dose distribution within deep tissues when the bolus materials were water, PLA, polystyrene, silica-gel or glycerol in each model, which is of great significance to protect organs at risk.

In the case of general superficial tumors represented by the S-T models, the dose distributions in deep tissues vary when using different materials as bolus during electron beam therapy. The analysis of the basic physical parameters of different materials in Table 2 indicates that the RD beyond *d*_max_ is positively correlated with the effective atomic number and negatively correlated with the electron density. Among these factors, the effective atomic number seems to play a more significant role. However, due to limited data, a specific relationship cannot be conclusively determined at this time. Nevertheless, based on the available information, it can be inferred that polystyrene emerges as the most suitable bolus material for the protection of deep tissues among the five materials considered. In postoperative breast cancer radiotherapy, utilizing the P-B model, the choice of bolus materials significantly impacts the dose distribution in lung tissue. Analysis based on the data presented in Table 2 suggests that the RDs in lung tissue are negatively correlated with electron density. Consequently, for postoperative breast cancer radiotherapy using electron beams, the utilization of a glycerol bolus results in the lowest dose in lung tissue, making it the most ideal material for protecting lung tissue among the five materials investigated in this study. As the effectiveness and control rates of tumor treatment continue to improve, the survival time of patients is increasing. Reducing the dose outside the target area has significant implications for minimizing radiation therapy side effects and lowering the incidence of radiation-induced secondary tumors. Radiation carcinogenesis falls under non-deterministic effects, and although the precise relationship between its incidence and radiation dose remains undetermined, numerous studies suggest that a higher radiation dose correlates with an increased incidence of radiation carcinogenesis [24, 25]. When utilized as bolus materials in electron beam radiotherapy for general superficial tumors and postoperative breast cancer, polystyrene and glycerol demonstrate effectiveness in reducing the dose to normal tissues. Although clinical data supporting this are currently lacking, we posit that this dose reduction could potentially yield greater benefits for patients undergoing radiotherapy.

Currently, there is a wide range of materials utilized for bolus production, and as materials science advances, it is expected that more suitable materials will become available for clinical use. While considering these materials, it is essential to take into account not only their ability to reduce air gaps between the bolus and the skin but also their impact on the dose distribution within the tissues. Kim *et al*. conducted a comparative analysis, examining variations between measurements during electron beam radiotherapy and Treatment Planning System (TPS, Pinnacle 9.2) calculations of superficial dose across diverse scenarios [26]. Their findings suggested that the thickness of the bolus could indeed impact the calculation error of superficial dose. Although the literature did not specify the bolus material and its influence on deep tissue dose, it indirectly substantiates that differences in bolus thickness contribute to dose disparities, aligning with the outcomes of our study. Zhang *et al*. [27] utilized the Monte Carlo method to compare the stopping power ratio of soft tissue with wax and water. Their assessment of dose differences under the bolus, particularly when wax was used as the equivalent medium for soft tissue, indicates that different bolus materials exhibit variations in radiation attenuation, subsequently affecting dose deposition in tissues. Nasrollah Jabbari and Hamid Reza Khalkhali investigated the relationship between shallow dose and bolus thickness in electron beam radiotherapy using the Monte Carlo algorithm [28]. Their findings indicated that high-density materials can more effectively increase shallow dose, providing evidence that bolus materials and thickness have distinct effects on tissue dose. Despite the similarities in results between our study and theirs, differences in research objectives and models prevent a systematic comparison. The observed difference between the two studies when the bolus material is polystyrene may be attributed to discrepancies in PSFs.

This study exclusively focused on investigating the impact of bolus material on the dose of deep tissue along the central axis of electron beam radiotherapy. It is acknowledged that variations exist between the dose at different depth planes and the central axis dose. Thus, for a comprehensive evaluation of the effect of bolus material on deep tissue dose, a thorough comparison of doses at various depth planes is imperative. The electron beam PSFs available on the IAEA official website were utilized in this study. Given the varying energy spectra of electron beams produced by different medical electron linear accelerators, differences may arise in how bolus materials affect the corresponding electron beam dose deposition. It is precisely for this reason, the simulation results were not directly cross-referenced with the measurement data and TPS calculations, thereby somewhat constraining the immediate clinical application of our research outcomes. In clinical practice, it is imperative to conduct experiments with the bolus material tailored to the specific equipment within the department. Furthermore, given the differences in the composition of various human body regions and the relatively simplified model used in this study, comprehending the impact of bolus materials on deep tissue dose is recognized as potentially more intricate. Regrettably, due to constraints within our experimental conditions, this study could not comprehensively address these intricate complexities.

## CONCLUSIONS

Selecting the suitable bolus material for different treatment scenarios can effectively minimize the dose to deep tissues. Among the five materials studied in this research, with a focus on protecting deep tissue, polystyrene and glycerol are deemed the most ideal bolus materials for general superficial tumor and postoperative breast cancer electron beam therapy, respectively.

## CONFLICT OF INTEREST

The authors declare that there is no conflict of interest regarding the publication of this paper.

## Funding

Wuxi Taihu Lake Talent Plan, Supports for Leading Talents in Medical and Health Profession; Scientific Research Project of Jiangsu Maternal and Child Health Association (FYX202016); Maternal and Child Health Research Project of Jiangsu Commission of Health (F202009); Scientific Research Project of Wuxi Commission of Health (M202041); Project plan of Wuxi Institute of translational medicine (LCYJ202210); Youth Scientific Research Project of Wuxi Municipal Health and Health Commission (Q201941).

## Data availability

The original data used to support the findings of this study are available from the corresponding author upon request.
